# The Influence of Conscious and Unconscious Body Threat Expressions on Motor Evoked Potentials Studied With Continuous Flash Suppression

**DOI:** 10.3389/fnins.2018.00480

**Published:** 2018-07-16

**Authors:** Tahnée Engelen, Minye Zhan, Alexander T. Sack, Beatrice de Gelder

**Affiliations:** Department of Cognitive Neuroscience, Faculty of Psychology and Neuroscience, Maastricht University, Maastricht, Netherlands

**Keywords:** motor evoked potentials, continuous flash suppression, emotion body perception, unconscious emotion perception, action preparation

## Abstract

The observation of threatening expression in others is a strong cue for triggering an action response. One method of capturing such action responses is by measuring the amplitude of motor evoked potentials (MEPs) elicited with single pulse TMS over the primary motor cortex. Indeed, it has been shown that viewing whole body expressions of threat modulate the size of MEP amplitude. Furthermore, emotional cues have been shown to act on certain brain areas even outside of conscious awareness. In the current study, we explored if the influence of viewing whole body expressions of threat extends to stimuli that are presented outside of conscious awareness in healthy participants. To accomplish this, we combined the measurement of MEPs with a continuous flash suppression task. In experiment 1, participants were presented with images of neutral bodies, fearful bodies, or objects that were either perceived consciously or unconsciously, while single pulses of TMS were applied at different times after stimulus onset (200, 500, or 700 ms). In experiment 2 stimuli consisted of neutral bodies, angry bodies or objects, and pulses were applied at either 200 or 400 ms post stimulus onset. In experiment 1, there was a general effect of the time of stimulation, but no condition specific effects were evident. In experiment 2 there were no significant main effects, nor any significant interactions. Future studies need to look into earlier effects of MEP modulation by emotion body stimuli, specifically when presented outside of conscious awareness, as well as an exploration of other outcome measures such as intracortical facilitation.

## Introduction

Perceiving threat cues from others will likely trigger a fight, flight or freeze response in the observer, supporting the notion that emotion and action readiness are tightly linked (Frijda, 2010; Eder and Rothermund, 2013). This action readiness is reflected in physiological responses such as changes in heart rate, pupil dilation, skin conductance, and muscle activations (Bradley et al., 2008; Roelofs et al., 2010; Huis In ’t Veld et al., 2014), and is also evident in the state of the cortical motor system(e.g., de Gelder et al., 2004; Goldberg et al., 2014). The current study set out to explore if and when stimuli portraying whole body postures of emotion alter motor cortex excitability, and whether conscious perception of such stimuli influences this modulation.

One direct way of assessing the state of the motor system is by evaluating corticospinal excitability (CSE), which can be measured by calculating the amplitude of motor evoked potentials (MEPs). MEPs are elicited with single pulses of transcranial magnetic stimulation (TMS) to M1, and they can be measured from the targeted muscle using electromyography (EMG). Under conditions of conscious perception, emotion related modulations of MEP amplitude were demonstrated in response to emotional scenes. Studies examining responses to either pleasant or unpleasant pictures taken from the International Affective Picture System (IAPS) have shown increased MEP amplitude for affective versus neutral scenes (Hajcak et al., 2007; Coombes et al., 2009; Coelho et al., 2010; van Loon et al., 2010). Likewise, modulation of MEP amplitude in response to social threat has been demonstrated by comparing the response to fearful faces to either neutral or happy faces. Again, an increase in MEP amplitude in response to fearful faces compared to both other conditions was observed (Schutter et al., 2008).

Similarly, studies have also explored emotion-dependent MEP modulations in response to whole body expressions of emotion, however, with mixed results. Initial work showed that at 300 ms post stimulus onset, both pictures of emotional (joy or fear) and neutral movements elicited greater MEP amplitude compared to neutral static postures, suggesting in this case it might be implied motion, rather than valence, modulating MEP amplitude (Borgomaneri et al., 2012). On the other hand, not only implied motion, but also social intentions seem to be of importance. Bucchioni et al. (2013) showed for example that MEP amplitude is enhanced when observing an actor passing a ball to another actor, rather than throwing the ball at a wall, suggesting the motor system certainly codes for more than simply the amount of perceived motion within an action. Recent work by Hortensius et al. (2016) found increased MEP amplitude in response to dynamic angry whole body movements at 300 ms post stimulus onset compared to fearful and neutral movements, irrespective of whether the direction of the movement was toward or away from the observer. Further experiments looking at whole body expressions of emotion have shown an increase in MEP amplitude in response to fearful compared to neutral actions as early as 70–90 ms post stimulus onset (Borgomaneri et al., 2015a). Yet, another experiment found a reduction in MEP amplitude of emotional versus neutral bodies at 150 ms, whereas a general increase in MEP amplitude for all stimuli was observed at 300 ms (Borgomaneri et al., 2014). When also taking into account intracortical facilitation (ICF) and inhibition (ICI), it was found that at 125 ms post stimulus onset ICF was suppressed specifically for fearful bodies, whereas this effect was absent for both MEPs and ICI (Borgomaneri et al., 2015b). This early suppression effect of ICF in response to fearful bodies was later replicated, and found to be related to individual Behavioral Inhibition System (BIS) sensitivity (Borgomaneri et al., 2017). The current study aimed to clarify whether MEPs are indeed modulated by emotional body expressions by assessing MEP amplitude at several time points after stimulus onset in response to both fearful and angry whole-body expressions of emotion.

The aforementioned studies demonstrate that motor cortex excitability can be modulated by the presence of social threat represented by whole body expressions of emotion. So far, experiments have focused on MEP modulation in response to consciously perceived threat. However, research suggests that salient signals, such as threat, have the ability to be processed also when presented outside of visual awareness. Ample evidence on the possibility of unconscious processing of emotions results from the study of a phenomenon called affective blindsight. Affective blindsight can occur in patients that are cortically blind as a result of lesions to their primary visual cortex. Despite being blind in part of their visual field, they are still capable of judging in their blind visual field whether a face is expressing happy or fear above chance level, without having any conscious percept of the stimulus (de Gelder et al., 1999). Unconscious processing of emotional information is not unique to these patients, however. By applying TMS to primary visual cortex to disrupt visual awareness, Jolij and Lamme (2005) were able to mimic affective blindsight in healthy participants.

Importantly, processing of unconscious emotional information has an effect on the observer, both on a physiological and a behavioral level. On the physiological level, unconsciously perceived emotions, expressed through either the body or the face, have been shown to cause changes in skin conductance (Esteves et al., 1994; Williams et al., 2004), pupil dilation (Tamietto et al., 2009, 2015), heart rate (Ruiz-Padial et al., 2005, 2011), heart rate variability (Bulut et al., 2018), evoking of startle reflexes (Hamm et al., 2003), levels of stress hormone (Van Honk et al., 1998), and have been shown to change EMG responses measured from facial muscles in both brain damaged patients (Tamietto et al., 2009) and in healthy participants (Dimberg et al., 2000). On the behavioral level, masked fearful faces have been shown to modulate orienting of covert spatial attention (Carlson and Reinke, 2008), the recognition of unmasked happy faces gets delayed by simultaneously presented masked fearful face (Tamietto and de Gelder, 2008), and suppressed angry faces can influence the likeability of novel items (Almeida et al., 2013), to name a few examples.

Several studies have looked into the level of processing of unconsciously perceived emotion in the brain, many of which have demonstrated the crucial role of subcortical areas. Subcortical areas that have been highlighted in multiple studies include superior colliculus, amygdala, and pulvinar (Tamietto and de Gelder, 2010). Yet cortical regions have equally been implicated as playing a role in processing of unconscious emotional signals. For example, one fMRI study involving a patient suffering from visual extinction, following lesions in the parietal cortex, found that presenting fearful body postures in the affected hemifield resulted in activations in striate and extrastriate visual areas, as well as activity in posterior cingulate cortex (Tamietto et al., 2015). Another fMRI study measured responses to angry and neutral whole body expressions presented in the blind field of a cortically blind patient. When contrasting unconsciously perceived angry and neutral bodies, they found widespread cortical activity, which included somatosensory, motor and premotor cortices (Van den Stock et al., 2011).

In general, similar to consciously perceived threat, it seems that unconsciously perceived threat might relate to action preparation in a similar way, as is suggested by for example changes in heart rate (Ruiz-Padial et al., 2005, 2011), or findings of motor cortex activity in response to angry bodies (Van den Stock et al., 2011). The question remains, however, if these unconscious brain responses to social threat extend to changes in excitability of the motor system, which provide a direct measure of action preparation. In addition, previously used methodology to study this topic (fMRI and most physiological measures), lack the ability to draw any conclusions about timing of such preparatory responses. This current study therefore aimed to answer the question whether unconsciously perceived whole body expressions of threat can also prime action readiness in M1, and the timing of when such excitability changes would occur. In order to accomplish this, we employed the continuous flash suppression (CFS) paradigm (Tsuchiya and Koch, 2005). CFS is a method whereby stimuli are rendered invisible to the participant by suppression of one image through interocular competition. Suppression is accomplished by presenting one eye with a dynamic colorful mask at a 10 Hz flickering frequency, while the other eye is presented with a stimulus of interest, but with lower contrast. The result is a reliable suppression of the conscious percept of the stimulus of interest.

A previous breaking from continuous flash suppressions (b-CFS) experiment established that suppression times for body stimuli were shorter for angry compared to both neutral and fearful postures, whereas fearful postures had longer suppression times compared to both other categories (Zhan et al., 2015). Despite fear and anger both conveying signals of threat, neuroimaging results suggest they might not be processed in the exact same way, as threat conveyed by an anger signal is more direct and less ambiguous compared to fear expressions (Pichon et al., 2009). Therefore, we decided to explore separately the effects of fear and angry body postures on motor cortex excitability.

Thus far, no previous studies have looked into modulation of MEPs in response to unconsciously perceived emotion stimuli. Feasibility of inducing changes in motor cortex excitability through masked stimuli has been demonstrated (Théoret et al., 2004a), but to date very few studies have used MEP amplitude as an outcome measure combined with presentations of stimuli outside of awareness. One study assessed MEP amplitude in response to masked self-images, and found a significant increase in MEP amplitude in response to self- compared to other-faces (Théoret et al., 2004b). On the other hand, a study using a masked priming paradigm that investigated the effect of implied action images of hands found results for action versus still hands, but only when the stimuli were presented supraliminally (Mattiassi et al., 2014). Although bodily action stimuli in general have been shown to modulate CSE (Fadiga et al., 2005), they do not carry the same saliency and relevance as emotionally laden actions, which could explain why their influence on M1 is restricted to conscious perception.

The goal of the current experiment was twofold; firstly, we aimed to replicate previous findings that consciously perceived, threat related body postures, compared to neutral postures, may alter the state of the motor cortex. Secondly, we wanted to explore whether such modulations were also present in unconscious perception of threatening body postures. In experiment 1, participants performed a CFS task in which they had to indicate after each trial whether a stimulus was consciously perceived or not. The stimulus categories presented were either fearful bodies, neutral bodies (standing still), or objects (lamps). To manipulate stimulus visibility, target images were presented to either the suppressed eye only, or both eyes, resulting in unseen or seen percepts. During task performance, single TMS pulses were administered to the hand hot-spot of left M1 while MEPs were measured from the first dorsal interosseous (FDI) muscle of the right hand. TMS pulses were triggered at three different time points, either at 200, 500, or 700 ms post stimulus onset. The design of experiment 2 was similar to that of experiment 1, with the exception that stimulus categories consisted of either angry bodies, neutral bodies, or objects, while TMS pulses were triggered at either 200 or 400ms post stimulus onset. We hypothesized that changes of MEP amplitude would be specific to the stimulus categories portraying threat (fear and anger), and to the early time points, both in the consciously and unconsciously perceived threat situations.

## Materials and Methods

### Participants

Twenty-one healthy volunteers [15 female, mean age (*SD*) = 24.5(3.3)] participated in experiment 1, and 30 healthy volunteers [23 female, mean age (*SD*) = 21(3.2)] participated in experiment 2. All participants were right handed and had normal or corrected to normal vision. If a participant had corrected to normal vision, they were asked to wear contact lenses during the experiment to allow for the wearing of the prism glasses (see task). All participants were unaware of the goal of the study until after the completion of the experiment. Before the start of the experimental proceedings, participants provided written informed consent and were screened for TMS safety based on published safety guidelines (Rossi et al., 2012). The study was performed in accordance to the Declaration of Helsinki and approved by the local ethical committee.

### Stimuli

The stimuli were selected from a validated set of body images, previously used by Stienen and de Gelder (2011). Stimuli consisted of static, gray-scaled body images portraying either neutral (standing still) or fearful postures in experiment 1, or neutral and angry postures in experiment 2. These postures were portrayed by both male and female actors, with their facial information removed. In total 10 actor identities (5 females) were used. As a control condition gray-scale images of lamps were used (Stein et al., 2012). The stimuli spanned within 1.8° × 4.3° visual angles.

### Continuous Flash Suppression Task

During the experiment, the participants performed a CFS task for which the parameters were as follows; the task was presented with MATLAB (MathWorks, Natick, MA, United States) using Psychtoolbox (Brainard, 1997). The task background was set to gray (RGB value = 128,128,128), on which two rectangles (240 pixels × 160 pixels, 6.4° × 4.27° visual angle) were placed side by side at the center of the screen, 286 pixels apart from one another (visual angle = 6.67°), and with a black fixation cross in the center of each rectangle. A 10-pixel frame delineated the border of the rectangles. To allow for the presentation of different stimuli to each of the eyes, participants wore prism glasses (diopter = 8), ensuring that the perception of each rectangle was shifted back to the center of the screen (method as described in Schurger, 2009). A cardboard divider was placed between the screen and the chinrest, dividing the screen into two halves, which ensured that each eye would not receive information from the contralateral side of the screen.

**FIGURE 1:**
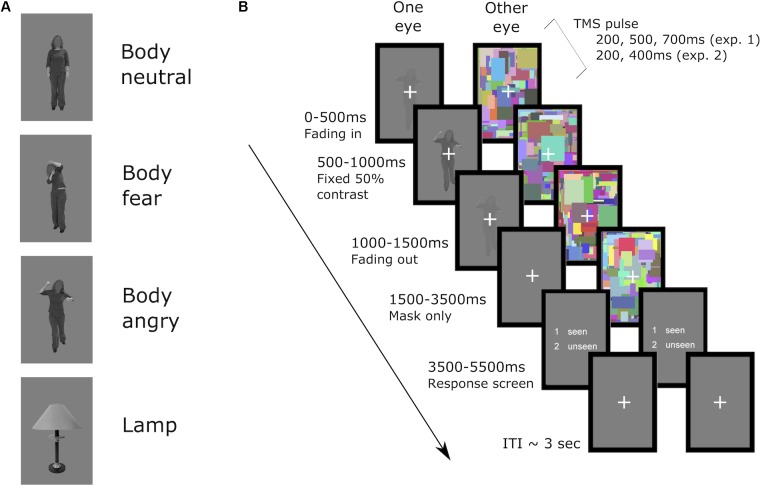
**(A)** examples of the stimuli used in both experiments and **(B)** an example of an unseen trial. First, in the suppressed eye the stimulus was faded in up to 50% contrast in the first 500 ms, then presented at 50% contrast for 500 ms, and in the subsequent 500 ms faded out. The dynamic noise was presented in the other eye from the start of the trial until 2000 ms after stimulus offset in the suppressed eye. Then a response screen, presented to both of the eyes, appeared for 2000 ms in which the participant replied whether any stimulus was seen or unseen via button press. A fixation cross was then presented to both eyes until the start of the next trial (ITI on average of 3.5 s). Single pulses of TMS were applied at a fixed interval after the onset of the fading in of the stimulus, either at 200, 500, or 700 ms in experiment 1 or 200, or 400 ms in experiment 2. Seen trials were identical in timing, with the only difference that the stimulus of interest was presented to both of the eyes, overlaid on top of the dynamic noise in the non-suppressed eye.

During each trial, dynamic noise images (160 pixels × 240 pixels, flashing at 10 Hz) were presented in one of the two rectangular frames in a counterbalanced manner. The noise images consisted of overlapping and colorful small rectangles (height and width within 2°), and were drawn randomly from 600 unique noise images. The target stimulus was projected into the other rectangular frame. For the seen trials the target stimulus was overlaid on the noise images, and thus was presented to both eyes. The duration of the stimulus presentation was set to 1.5 s, and consisted of gradually ramping up of the stimulus contrast from 0 to 50% contrast in the first 500 ms in five steps (100 ms per step), then presentation of the stimulus at 50% contrast for another 500 ms, and lastly ramping down stimulus contrast back to 0%. The dynamic noise images were presented for 2 more seconds after the offset of the target stimulus, to ensure no afterimage of the target was perceived. At the end of each trial a response screen (2 s) was presented, during which the participant had to respond with their left hand whether a stimulus (body or lamp) had been perceived or not. To rule out any possible contamination of the EMG signal in the right hand caused by response preparation in contralateral motor cortex, the two answer keys presented in the response screen were randomly switched in a counterbalanced manner, and participants were instructed to not respond until the response screen appeared. Trials in which the response fell outside of the 2 s response window were excluded from analysis. A jittered ISI of 2.5, 3, 3.5, 4, or 4.5 s followed the offset of the response window. See **Figure 1** for an example trial of the task.

### TMS Stimulation and EMG Recording

During the performance of the CFS task, single pulse TMS (spTMS) was applied over the M1 hand hotspot in the left hemisphere at 120% resting motor threshold (mean stimulation intensity 45% MSO in experiment 1, and 42% MSO in experiment 2) using a MC-B70 figure-of-eight coil and Magpro X100 stimulator (Medtronic Functional Diagnostics A/S, Skovlunde, Denmark). The coil was placed tangentially to the scalp with the handle pointing backward at an angle of 45 degrees from the midline. Motor threshold of each participant was determined as the lowest stimulation intensity needed to evoke an MEP (>0.05 mV) in 5 out of 10 pulses, while the right hand was at rest. For both experiments, the inter-pulse interval was jittered around 9 s, and the timing of the pulse was either 200, 500, or 700 ms post stimulus onset in experiment 1, and 200 or 400 ms in experiment 2. One pulse was applied randomly at one of the timings per trial. EMG of the right FDI muscle was recorded for 100 ms pre- until 150 ms post TMS pulse. Pre-gelled silver-chloride disposable surface electrodes were placed in a belly-tendon montage with a ground electrode on the wrist. The EMG signal was recorded using a Powerlab 4/35 data acquisition device with a Bio Amp system (ADInstruments, Sydney, Australia). The signal was amplified, sampled at 4 k/s, band-pass filtered (20–2000 Hz), digitized and stored on a computer for offline analysis.

### Procedure

Each participant came in for one 2-h session. Participants were seated in front of an LCD computer screen used for presentation of the task (Iiyama prolite B2481HS, 24″, resolution 1920 × 1080). First, each participant had a short practice run to ensure they could stably merge the two rectangles, and that suppression of the stimuli was successful. Electrodes for the EMG recordings were placed on the right hand, and it was checked that noise levels were below 0.02 mV. An optimal location for stimulation was found by moving the TMS coil over left motor cortex and observing the peak-to-peak amplitudes of MEPs. Once a location was found that elicited the largest peak-to-peak MEP amplitude for the stimulation of the FDI muscle, the coil was fixed into position using a coil holder, while the participant rested their head in a chinrest. Participants were instructed to sit as still as possible during the rest of the session. To ensure that no shifts in coil position occurred, the MEP amplitudes were monitored throughout the session and adjustments were made by the experimenter if necessary. In experiment 2, in order to try and reduce MEP variability, Neuronavigation was used and the location of the hotspot was marked on a template brain to ensure more stability of the stimulation site (BrainVoyager TMS Neuronavigator software, Brain Innovation, Maastricht, Netherlands). During each run participants were asked to keep their right hand as relaxed as possible. They were additionally instructed to fixate and free-fuse the two rectangles into one. The experiment only started after participants reported that they could clearly see only one rectangle, and that this view was stable. All participants reported successful and stable fusion. A total of 4 runs of the task were performed, each run containing 90 trials and lasting around 13 min. A total of 360 trials were acquired for each participant, half of which were seen and half of which were unseen. In experiment 1, for each of the 18 conditions [2 visibility (seen/unseen) × 3 stimulus (body fear/body neutral/lamp) × 3 TMS timing (200 ms/500 ms/700 ms)] 20 trials were presented. In experiment 2, each of the 12 conditions [(2 visibility (seen/unseen) × 3 stimulus (body anger/body neutral/lamp) × TMS timing (200 ms/400 ms)] was presented for 30 trials.

### Data Preprocessing

Neurophysiological and behavioral data were processed offline. All trials that were incorrect (i.e., unseen trials that were reported as seen and vice versa) were removed from analysis to ensure only successfully suppressed trials were included (12.3% of trials in experiment 1, 12.6% of trials in experiment 2). Mean MEP peak-to-peak values were calculated in mV and outliers were removed that deviated more than 2.5 SDs from the mean of each condition (1.7% of trials in experiment 1, 2.1% of trials in experiment 2). To rule out any effects of muscle pre-contraction on MEP amplitude, the highest peak-to-peak value from the 100 ms preceding the TMS pulse were used to exclude all trials with a deviation of more than 3 SDs of the mean in each run, as the noise on the EMG signal could sometimes vary between runs (1.6% of trials in experiment 1, 1.7% of trials in experiment 2). One participant from experiment 1 and one participant from experiment 2 were excluded from the analysis as their overall average MEP peak to peak amplitude was an outlier with a deviation larger than 2.5 standard deviations from the group mean.

### Analysis

For experiment 1, a 2 × 3 × 3 repeated-measures (RM) ANOVA was used to investigate the effects of stimulus visibility (seen/unseen) of different stimulus conditions (body fear/body neutral/lamp) on MEP amplitude at three different time points (200 ms/500 ms/700 ms). For experiment 2, a 2 × 3 × 2 RM ANOVA was used to investigate the effects of stimulus visibility (seen/unseen) of different stimulus categories (body angry/body neutral/lamp) on MEP amplitude at two different time points (200ms/400ms). As the assumption of sphericity was violated for the data in experiment 1, Greenhouse-Geisser corrected *p*-values are reported. Additionally, a Bayesian repeated measures ANOVA was run for each experiment using the JASP software package [JASP Team (2018). JASP (Version 0.8.6); Rouder et al., 2012; Morey and Rouder, 2015].

## Results

### Results Experiment 1

A 2 × 3 × 3 RM ANOVA showed a significant main effect of the time of the pulse [*F*(1.360,25.832) = 10.593, *p* = 0.001, $\eta_{\text{p}}^{2}$ = 0.358]. This main effect was driven by a significant difference between 200 and 500 ms (*p*_bonf_ = 0.003), and a significant difference between 200 and 700 ms (*p*_bonf_ = 0.012). There was no main effect of visibility [*F*(1,19) < 0.001, *p* = 0.990, $\eta_{\text{p}}^{2}$= 0.000], nor a main effect of stimulus condition [*F*(1.954,37.126) = 0.228, *p* = 0.792, $\eta_{\text{p}}^{2}$ = 0.012], nor any interactions between either visibility and stimulus condition [*F*(1.945,36.962) = 1.852, *p* = 0.172, $\eta_{\text{p}}^{2}$ = 0.089], visibility and pulse time [*F*(1.942,36.894) = 2.197, *p* = 0.127, $\eta_{\text{p}}^{2}$ = 0.104], stimulus condition and pulse time [*F*(2.441,46.382) = 0.921, *p* = 0.422, $\eta_{\text{p}}^{2}$ = 0.046], or a visibility × stimulus condition × pulse time interaction [*F*(3.321,63.100) = 1.728, *p* = 0.165, $\eta_{\text{p}}^{2}$ = 0.083].

A Bayesian repeated measures ANOVA using default prior scales revealed that a model with the main effect of pulse time was preferred, with a Bayes factor of 2.143e+6, providing decisive evidence for the alternative hypothesis (Wetzels et al., 2011). For the full results of the Bayesian RM ANOVA, including analysis of effects across matched models see Supplementary Material.

**FIGURE 2:**
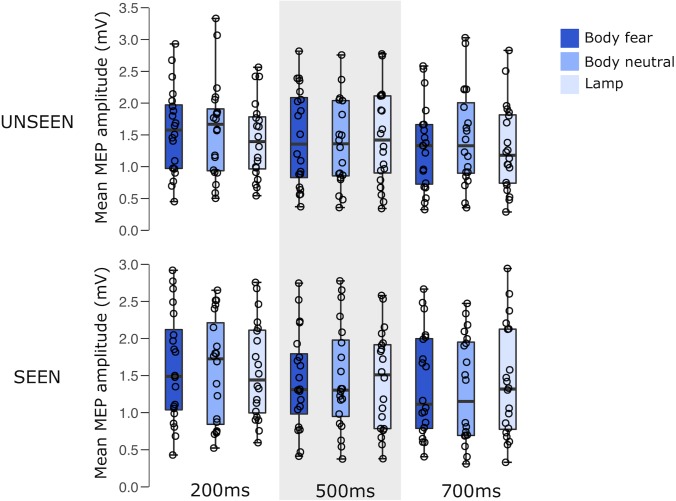
Boxplots representing the observed MEP amplitude in mV for the different conditions in experiment 1. Circles reflect the individual participant average corresponding to each condition.

For an overview of the results of experiment 1 see **Figure 2** and **Table 1**.

**Table 1:** Mean MEP amplitude (SD) for the different conditions in experiment 1.

	Unseen			Seen		
	200 ms	500 ms	700 ms	200 ms	500 ms	700 ms
Body fear	1.57 (0.68)	1.46 (0.76)	1.31 (0.67)	1.61 (0.75)	1.43 (0.66)	1.38 (0.71)
Body neutral	1.59 (0.76)	1.38 (0.69)	1.46 (0.77)	1.58 (0.73)	1.47 (0.72)	1.30 (0.72)
Lamp	1.43 (0.61)	1.46 (0.74)	1.30 (0.69)	1.57 (0.66)	1.44 (0.67)	1.41 (0.76)

### Results Experiment 2

A 2 × 3 × 2 RM ANOVA showed no main effects for either visibility [*F*(1,28) = 2.607, *p* = 0.118, $\eta_{\text{p}}^{2}$ = 0.085], stimulus condition [*F*(2,56) = 1.002, *p* = 0.374, $\eta_{\text{p}}^{2}$ = 0.035], or pulse time [*F*(1,28) = 1.133, *p* = 0.296, $\eta_{\text{p}}^{2}$ = 0.039]. There were also no interactions between either visibility and condition [*F*(2,56) = 1.297, *p* = 0.281, $\eta_{\text{p}}^{2}$ = 0.044], visibility and pulse time [*F*(1,28) = 0.180, *p* = 0.675, $\eta_{\text{p}}^{2}$ = 0.006], condition and pulse time [*F*(2,56) = 0.311, *p* = 0.734, $\eta_{\text{p}}^{2}$ = 0.011], or a visibility × stimulus condition × pulse time interaction [*F*(2,56) = 0.387, *p* = 0.681, $\eta_{\text{p}}^{2}$ = 0.014].

The results of the Bayesian repeated measures ANOVA analysis showed that none of the included models were able to outperform the null model. The analysis of effects across matched models revealed that all models of the main effects or interactions had an inclusion Bayes factor ranging between 0.070 and 0.519, providing either anecdotal or decisive evidence for the null hypothesis. For the full table of results of the Bayesian RM ANOVA see the Supplementary Material.

**FIGURE 3:**
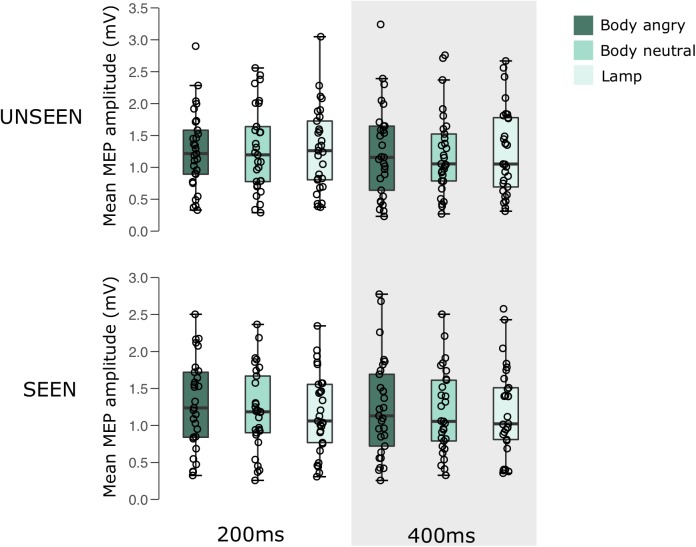
Boxplots representing the observed MEP amplitude in mV for the different conditions in experiment 2. Circles reflect the individual participant average corresponding to each condition.

For an overview of the results of experiment 2 see **Figure 3** and **Table 2**.

**Table 2:** Mean MEP amplitude (SD) for the different conditions in experiment 2.

	Unseen		Seen	
	200 ms	400 ms	200 ms	400 ms
Body angry	1.27 (0.61)	1.25 (0.72)	1.29 (0.61)	1.24 (0.66)
Body neutral	1.29 (0.67)	1.22 (0.65)	1.21 (0.56)	1.18 (0.56)
Lamp	1.29 (0.66)	1.25 (0.68)	1.17 (0.54)	1.20 (0.60)

## Discussion

The current experiment set out to explore whether viewing emotional body postures can modulate the excitability of motor cortex during both conscious and unconscious perception. Specifically, in experiment 1 we compared the amplitude of MEPs for conscious and unconscious viewing of either neutral or fearful bodies, and lamps as control stimuli, at different time points after stimulus onset (200, 500 and 700 ms) in a CFS task. Whereas there seemed to be a general modulation of MEP amplitude depending on the time point of the stimulation, no clear effect of the presented stimulus was apparent when the stimulus was unconsciously or consciously perceived. In experiment 2, we again compared MEP amplitudes in responses to consciously or unconsciously perceived bodies and lamps, this time investigating the effect of anger, and focusing on the time points 200 and 400 ms post stimulus onset. There were no significant effects of the different presented stimuli, either in the conscious or unconscious conditions, nor an effect of the timing of the pulse. Below we will discuss several factors that might have contributed to the null findings of the current study.

### Not Only the “Where”, but Also the “When” of Action Preparation

A main advantage of TMS is its high temporal resolution, and using this method to study motor cortex reactivity to threat can give valuable insight into the exact timing of preparatory responses. So far, several studies have tried to address this question. Their results suggest that these responses can occur already at very early stages of processing. Significant differences in motor cortex excitability between emotional and neutral body postures have been shown to occur as early as 70–90 ms post stimulus onset (Borgomaneri et al., 2015a), but also at 100–125 ms (Borgomaneri et al., 2015b, 2017), and 150 ms (Borgomaneri et al., 2014). These findings are in line with MEG results showing emotion specific effects for body stimuli in the dorsal stream for action as early as 80 ms post stimulus onset (Meeren et al., 2016). When looking at later time points, specifically 300 ms, effects seem to be driven by actions in general rather than being emotion specific (Borgomaneri et al., 2012). On the other hand, Hortensius et al. (2016) found an effect of anger compared to both fear and neutral at 300 ms. In the current study, we used the time points 200, 400, 500, and 700 ms. In light of previous work, there is a possibility that our lack of clear findings is the result of ‘missing’ the critical time window in which CSE changes occur. A key difference with previous work, however, is that given the CFS paradigm we used, it was crucial to use a fading in of the stimulus to full contrast to achieve stable suppression, whereas no such restriction exists in experiments that focus purely on conscious perception of emotional bodies. This makes it difficult to predict when effects of emotion on M1 would occur. So far, studies on unconscious emotion processing have employed methods such as fMRI and physiological recordings. Albeit interesting with regards to mapping how our brain and body respond to unconscious emotion processing, outcome measures like the BOLD signal, pupil dilation, heart rate or skin conductance are sluggish and lack the power to give any information on the chronometry of when our brain prepares for action in response to unconsciously perceived emotions. One study that did focus on timing of brain responses during unconscious emotion processing, used a combination of backward masking of fearful and neutral faces with EEG (Williams et al., 2004). They found evidence for distinct processing of fearful versus neutral faces within the first 400 ms of processing. More precisely, unconsciously perceived fear faces elicited a greater N2 component at 200 ms post stimulus onset, as well as a faster P1 component within 100 ms post stimulus onset. No effects for unconsciously perceived faces were found in later components, in contrast to the conscious fearful faces that still modulated the N4 component peaking around 400 ms, suggesting that unconscious emotion stimuli might not be processed beyond an early alerting function.

In the current study, we were unable to find any effects of unconsciously presented emotion body stimuli on the motor system at any of the tested time points. As there are thus far no other studies looking into when unconsciously perceived emotion stimuli influence the motor system, this lack of findings might result from probing M1 at the wrong time points.

### Differences in Fearful and Angry Body Postures as Signals of Social Threat

Despite the fact that each is related to threat, body postures conveying fear or anger do not necessarily evoke the same response. When perceiving another person in fear, this could reflect fear for something in the environment, or fear for the observer, whereas anger directed at the observer provides an unambiguous threat signal. In accordance with this, neuroimaging studies have shown that perception of fear or anger signals can have distinct neural signatures. Pichon et al. (2009) directly tested the differences in responses to fear versus angry dynamic bodies using fMRI. Whereas both angry and fearful dynamic bodies resulted in similar activations in areas like the amygdala, as well as temporal and prefrontal areas, angry bodies additionally activated anterior temporal regions, premotor cortex, ventro-medial prefrontal cortex and orbitofrontal cortex. These findings concur with the idea that compared to fear, anger signals more directly require behavioral adjustments from the observer. These differences between fear and anger also seem to hold when processed unconsciously, as b-CFS results show that whereas angry bodies break faster from suppression than neutral bodies, fear bodies show the opposite pattern (Zhan et al., 2015).

In our separate experiments, we tested the influence of both fearful and angry body postures on motor cortex excitability, and found that neither of the two social threat signals changed the amplitude of MEPs compared to neutral bodies or lamps. What could be of particular interest for future studies would be to test both social threat types within the same experiment, enabling direct comparisons of the two.

### CSE as a Marker of Action Preparation in the Face of Social Threat

Motor evoked potentials provide a read out of the current state of cortico-spinal excitability at the time that the TMS pulse is administered (Rothwell, 1997), and thus provide a reflection of the functional state of the motor system (Bestmann and Krakauer, 2015). One issue of using MEPs as the outcome measure of the state of M1, however, is that they provide a compound signal, reflecting not only direct activation (D-waves) of pyramidal tract neurons, but also indirect activation (I-waves) reflecting input onto cortico-spinal pyramidal tract neurons (Rothwell et al., 1999). One way of circumventing this problem is by means of paired pulse stimulation protocols, which allow for the measurement of intracortical facilitation (ICF) and short intracortical inhibition (SICI) (Kujirai et al., 1993; Ziemann et al., 1996). In these protocols, first a low-intensity conditioning pulse is administered, followed by a supra-threshold test pulse, and when these two pulses have certain time intervals (1–5 ms for SICI and 7–20 ms for ICF) this results in either facilitation or inhibition of the following MEP. ICF and SICI are thought to occur at the cortical level, and are thus not confounded by subcortical or spinal signals, and thereby provide a clean read out for the current state of M1.

So despite the fact that CSE as measured with MEPs has been shown to provide a marker for action preparation (Mars et al., 2007; van Elswijk et al., 2007), and MEP modulation in response to social threat has been demonstrated (Borgomaneri et al., 2014, 2015a), it is interesting to consider whether ICF and SICI could give a more comprehensive insight into how the motor cortex responds to threat. To date, three published studies have included ICF and SICI as a measure of responding to threatening bodies. Findings showed a reduction of the magnitude of ICF in response to fearful compared to neutral and happy bodies at 100–125 ms (Borgomaneri et al., 2015b), which was later replicated and shown to be larger for participants with a greater self-reported BIS score (Borgomaneri et al., 2017), but this ICF effect was absent at 70–90 ms (Borgomaneri et al., 2015a). No effects on SICI have been found so far. Interestingly, none of these studies included angry bodies as stimuli, which makes it worthwhile to consider how anger threat signals might affect intracortical facilitation and inhibition.

### MEPs as a Reliable Measure of CSE; Issues of Variability

Although widely used in both research and clinical settings, MEPs suffer from large intra- and inter-subject variability (Kiers et al., 1993). Many factors have been implicated in contributing to this variability, like pre-activation of the targeted muscle (Darling et al., 2006), site of the recording electrode (Dunnewold et al., 1998), intensity of the stimulation (Van der Kamp et al., 1996), state of action preparation (Klein-Flugge et al., 2013), and even gender and age (Pitcher et al., 2003). More recently, spontaneous intrinsic fluctuations of neural oscillatory activity have also been implicated in being a source of MEP variability (see e.g., Keil et al., 2014; Schulz et al., 2014; Guerra et al., 2016).

In the current study we tried to control for the influence of motor preparation in the contra-lateral hemisphere (responses were made using the left hand) by requiring a motor response in each trial, after a jittered amount of time, and randomizing the response buttons. Muscle pre-activation was accounted for by removing all trials that had a pre pulse EMG signal that exceeded a certain threshold. Nonetheless, the observed variability in the first experiment remained high. Therefore, in the second experiment we tried to reduce MEP variability by using navigated TMS, as this has been shown to increase stability of coil positioning (Cincotta et al., 2010). Nonetheless, when calculating the standard deviation for each participant based on all trials (without preprocessing), and then comparing the overall standard deviation between experiment 1 and 2 using the Mann–Whitney test (to account for different sample sizes), we did not see a significant reduction in variability between the two experiments (average *SD* experiment 1 = 0.927, average *SD* experiment 2 = 0.926, *W* = 295, *p* = 0.928). This is in line with a previous study that systematically compared MEP variability with and without neuronavigation, and did not find significant differences in the coefficient of variance between these two conditions (Jung et al., 2010). So, despite the undeniable value that MEPs have in measuring excitability of M1, large variability in data still poses an issue, and possibly can contribute to unclear or even null findings.

### Level of Processing of Suppressed Stimuli

Despite its great merit as a method for suppressing visual stimuli from awareness, there are also questions surrounding CFS. Especially questions regarding to which extent suppressed stimuli are actually being processed are of crucial importance, with some accounts reasoning representations of such stimuli is fractioned (Moors et al., 2017). In this vein, the current CFS study is not the first to report a lack of findings in response to suppressed emotional images. For example, one study using CFS to study physiological responses to suppressed emotional stimuli found a typical modulation of skin conductance and post-auricular reflexes, but no modulation of heart rate or eye-blink reflexes (Tooley et al., 2017). Likewise, another study combining CFS with EEG found that while consciously perceived emotional faces did modulate the expected ERP components (N170/EPN and LPP), no such modulations were observed in the condition in which the emotional faces were suppressed from visual awareness (Schlossmacher et al., 2017). Yet another experiment found that while threatening images that were breaking from suppression altered skin conductance and spatial attention, this effect was absent for the properly suppressed stimuli (Hedger et al., 2015). Using a behavioral CFS experiment investigating the redundant target effect using whole body expressions of emotion we previously found a facilitation of incongruent emotional stimuli on reaction times, rather than facilitation of congruent emotional stimuli which is typically observed for both patient and backward masking studies (Zhan and de Gelder, 2018). This again suggests different mechanisms of suppression under CFS. In the current study, we presented stimuli at 50% contrast in order to accomplish successful suppression, as well as removing all trials that broke from suppression from the analysis. These stringent measures might have resulted in the suppressed images not being processed at all, or at low levels which would not induce changes in MEP amplitude. For future studies, it would be of interest to test different stimulus contrasts, as well as comparing suppressed stimuli to ‘blank’ trials, in which only the flickering noise mask is presented to one eye, while the other eye is not presented with anything.

### The Influence of Task Parameters on MEP Modulation

In the current study, we presented static images of bodies and objects within the setting of a CFS task. During this task, half of the trials included suppression of these images from awareness. Our critical outcome measure therefore was a rating of whether or not the participants experienced a conscious percept of these images. This also means, however, a possibly critical difference between our task and previous studies that did find modulation of MEP amplitude in response to whole body expressions of emotion, namely the explicit identification of the emotional content of the stimulus. Indeed, all previous studies included the recognition of the expressed emotion in the body stimulus, either by verbal report or button press (Borgomaneri et al., 2012, 2014, 2015a,b, 2017; Hortensius et al., 2016). Whether such task manipulations actually influence the state of the motor cortex has not been directly tested yet, although similar discrepancies between results originating from differences in task instructions can be observed in emotion sensitive regions such as the amygdala (de Gelder et al., 2012). It also has to be mentioned that the current study only presented images of still neutral postures, meaning that any observed differences between threatening postures and neutral bodies or objects could result from the amount of implied motion displayed in the threatening stimulus.

### Future Directions

Irrespective of the findings for influences of threatening postures on CSE in the current study, there are several directions to explore in future studies. First of all, it will be interesting to see whether assessing CSE at different, and specifically earlier, time points will reflect significant modulations of the state of the motor system. EEG findings on masked fearful faces reported results within the first 100 ms post stimulus onset, and no effects were found in components occurring later than 200 ms onset (Williams et al., 2004). Similarly, for consciously perceived emotional body postures, effects on CSE can occur as early as 70–90 ms post stimulus onset (Borgomaneri et al., 2015a). This makes a window of 100–200 ms post stimulus onset especially interesting to explore. In addition to this, it will be valuable to assess both fearful and angry postures within the same experimental design, to see if previously suggested differential effects when these signals of threat are consciously perceived also extend to unconscious processing. Lastly, to specifically explore effects of intracortical inhibition and facilitation in the motor system, future studies should compare outcomes of not only MEPs *per se*, but also include ICF and SICI as measures of interest.

## Ethics Statement

This study was carried out in accordance with the recommendations of the local ethics committee at the faculty of psychology and neuroscience (the ECP), at Maastricht University. The protocol was approved by the ECP. All subjects gave written informed consent in accordance with the Declaration of Helsinki.

## Author Contributions

TE, MZ, AS, and BdG designed the experiments. TE acquired data and analyzed the data. All authors contributed to the manuscript.

## Conflict of Interest Statement

The authors declare that the research was conducted in the absence of any commercial or financial relationships that could be construed as a potential conflict of interest.
