# Supplementary material for: The Influence of Conscious and Unconscious Body Threat Expressions on Motor Evoked Potentials Studied With Continuous Flash Suppression
Source: Front Neurosci. 2018 Jul 16;12:480. doi: 10.3389/fnins.2018.00480 (PMC6054979; doi:10.3389/fnins.2018.00480)
Supplement: Supplementary file 1 [file Table_1.PDF]

Results Bayesian Repeated Measures ANOVA experiment 1

Model Comparison

Models	P(M)	P(M|data)	BF_M	BF_{10}	error %
Null model (incl. subject)	0.053	3.753e −7	6.755e −6	1.000	
Visibility	0.053	5.963e −8	1.073e −6	0.159	0.855
Stimulus condition	0.053	1.753e −8	3.156e −7	0.047	1.021
Visibility + Stimulus condition	0.053	2.828e −9	5.090e −8	0.008	1.796
Visibility + Stimulus condition + Visibility ∗ Stimulus condition	0.053	4.328e −10	7.791e −9	0.001	1.399
Pulse time	0.053	0.804	73.926	2.143e +6	0.577
Visibility + Pulse time	0.053	0.138	2.879	367478.939	1.766
Stimulus condition + Pulse time	0.053	0.037	0.687	98031.010	4.656
Visibility + Stimulus condition + Pulse time	0.053	0.007	0.124	18297.462	2.196
Visibility + Stimulus condition + Visibility ∗ Stimulus condition + Pulse time	0.053	0.001	0.023	3338.100	3.237
Visibility + Pulse time + Visibility ∗ Pulse time	0.053	0.010	0.183	26783.663	1.252
Visibility + Stimulus condition + Pulse time + Visibility ∗ Pulse time	0.053	4.992e −4	0.009	1330.176	2.158
Visibility + Stimulus condition + Visibility ∗ Stimulus condition + Pulse time + Visibility ∗ Pulse time	0.053	9.534e −5	0.002	254.079	4.314
Stimulus condition + Pulse time + Stimulus condition ∗ Pulse time	0.053	0.002	0.035	5119.749	1.847
Visibility + Stimulus condition + Pulse time + Stimulus condition ∗ Pulse time	0.053	3.494e −4	0.006	931.058	4.230
Visibility + Stimulus condition + Visibility ∗ Stimulus condition + Pulse time + Stimulus condition ∗ Pulse time	0.053	5.909e −5	0.001	157.474	1.986
Visibility + Stimulus condition + Pulse time + Visibility ∗ Pulse time + Stimulus condition ∗ Pulse time	0.053	2.416e −5	4.348e −4	64.371	2.407
Visibility + Stimulus condition + Visibility ∗ Stimulus condition + Pulse time + Visibility ∗ Pulse time + Stimulus condition ∗ Pulse time	0.053	4.403e −6	7.926e −5	11.734	2.195
Visibility + Stimulus condition + Visibility ∗ Stimulus condition + Pulse time + Visibility ∗ Pulse time + Stimulus condition ∗ Pulse time + Visibility ∗ Stimulus condition ∗ Pulse time	0.053	2.559e −6	4.606e −5	6.819	5.242

Note. All models include subject.

Analysis of Effects

Effects	P(incl)	P(incl|data)	$BF_{Inclusion}$
Visibility	0.263	0.145	0.172
Stimulus condition	0.263	0.044	0.046
Pulse time	0.263	0.987	2.166e +6
Visibility ∗ Stimulus condition	0.263	0.001	0.182
Visibility ∗ Pulse time	0.263	0.011	0.073
Stimulus condition ∗ Pulse time	0.263	0.002	0.052
Visibility ∗ Stimulus condition ∗ Pulse time	0.053	2.559e −6	0.581

Note. Compares models that contain the effect to equivalent models stripped of the effect. Higher-order interactions are excluded. Analysis suggested by Sebastiaan Mathôt.

Results Bayesian Repeated Measures ANOVA experiment 2

Model Comparison

Models	P(M)	P(M|data)	BF_M	BF_{10}	error %
Null model (incl. subject)	0.053	0.494	17.603	1.000	
Visibility	0.053	0.258	6.258	0.522	1.016
Stimulus condition	0.053	0.034	0.643	0.070	0.627
Visibility + Stimulus condition	0.053	0.018	0.334	0.037	1.325
Visibility + Stimulus condition + Visibility ∗ Stimulus condition	0.053	0.003	0.056	0.006	3.175
Pulse time	0.053	0.111	2.240	0.224	1.265
Visibility + Pulse time	0.053	0.055	1.056	0.112	1.160
Stimulus condition + Pulse time	0.053	0.008	0.140	0.016	1.361
Visibility + Stimulus condition + Pulse time	0.053	0.004	0.075	0.008	2.554
Visibility + Stimulus condition + Visibility ∗ Stimulus condition + Pulse time	0.053	7.020e −4	0.013	0.001	5.183
Visibility + Pulse time + Visibility ∗ Pulse time	0.053	0.011	0.204	0.023	5.724
Visibility + Stimulus condition + Pulse time + Visibility ∗ Pulse time	0.053	7.996e −4	0.014	0.002	5.940
Visibility + Stimulus condition + Visibility ∗ Stimulus condition + Pulse time + Visibility ∗ Pulse time	0.053	1.246e −4	0.002	2.521e −4	2.727
Stimulus condition + Pulse time + Stimulus condition ∗ Pulse time	0.053	5.573e −4	0.010	0.001	2.578
Visibility + Stimulus condition + Pulse time + Stimulus condition ∗ Pulse time	0.053	3.230e −4	0.006	6.532e −4	4.982
Visibility + Stimulus condition + Visibility ∗ Stimulus condition + Pulse time + Stimulus condition ∗ Pulse time	0.053	5.935e −5	0.001	1.200e −4	6.706
Visibility + Stimulus condition + Pulse time + Visibility ∗ Pulse time + Stimulus condition ∗ Pulse time	0.053	5.758e −5	0.001	1.165e −4	12.151
Visibility + Stimulus condition + Visibility ∗ Stimulus condition + Pulse time + Visibility ∗ Pulse time + Stimulus condition ∗ Pulse time	0.053	9.642e −6	1.736e −4	1.950e −5	5.280
Visibility + Stimulus condition + Visibility ∗ Stimulus condition + Pulse time + Visibility ∗ Pulse time + Stimulus condition ∗ Pulse time + Visibility ∗ Stimulus condition ∗ Pulse time	0.053	1.488e −6	2.678e −5	3.009e −6	19.670

Note. All models include subject.

Analysis of Effects

Effects	P(incl)	P(incl|data)	$BF_{Inclusion}$
Visibility	0.263	0.336	0.519
Stimulus condition	0.263	0.065	0.070
Pulse time	0.263	0.179	0.221
Visibility ∗ Stimulus condition	0.263	0.004	0.171
Visibility ∗ Pulse time	0.263	0.012	0.201
Stimulus condition ∗ Pulse time	0.263	0.001	0.075
Visibility ∗ Stimulus condition ∗ Pulse time	0.053	1.488e −6	0.154

Note. Compares models that contain the effect to equivalent models stripped of the effect. Higher-order interactions are excluded. Analysis suggested by Sebastiaan Mathôt.
